# Electrical Tomography Reconstruction Using Reconfigurable Waveforms in a FPGA

**DOI:** 10.3390/s21093272

**Published:** 2021-05-10

**Authors:** Andres Vejar, Tomasz Rymarczyk

**Affiliations:** 1 Institute of Computer Science and Innovative Technologies, University of Economics and Innovation in Lublin, 20-209 Lublin, Poland; 2 Research & Development Centre Netrix S.A., 20-704 Lublin, Poland

**Keywords:** electrical tomography, FPGA, potential profile, tomographic imaging

## Abstract

The principal objective of this research is to conceive a mobile system based on electrical tomography for subsurface imaging and monitoring in order to enable simultaneous recording of electrical potentials of cardiac and pulmonary activity. For an exploration of excitation waveforms in electrical tomography, specialized hardware is required. As the main principle of tomography is the measurement of electrical perturbations on an unknown object, it is crucial to synchronize excitation and sensing processes in a very precise way for the purpose of acquiring meaningful data. To cope with this problem, an FPGA device is used, with an architecture that allows us to trigger excitation signals and to read sensed data simultaneously via independent processes that share the same clock. In this way, waveform reconfiguration on frequency and shape can be provided and studied. The system is connected to a standard microcontroller SoC with a simple API that allows for IoT capabilities for on-line operation and tracking, given that the design is targeted for in vivo medical monitoring. As a result of the research work, a measuring device was developed, the surface data analyzed and the image was reconstructed using the selected configuration.

## 1. Introduction

The main goal of the project, under which the results of this research work were presented, is to create a mobile tomographic system for 3D imaging and surface monitoring using body surface potential mapping. The system consists of a mobile device enabling simultaneous recording of electrical potentials of heart activity and lung ventilation. In the diagnosis of many disease entities (chronic respiratory and cardiovascular diseases in the early stages), it is necessary to monitor important vital signs of the patients continuously [1] during extended periods of time that can range up to several days. The future of medical diagnostics lies in devices that perform long-term patient monitoring [2], recording a wide diagnostic spectrum in order to detect target pathologies. The scope of monitoring can consider myocardial function, arrhythmias, blood flow, atrioventricular conduction disturbances and lung respiratory capacity. The latter can be tracked considering the changes in lung impedance via electrical tomography [3,4]. There are many tomographic solutions and methods for analyzing measurements or reconstructing images [5,6,7,8,9,10,11,12,13], sharing the fundamental principles but varying in the perturbation type, the surface information sensed, the geometry of the problem and the dynamics characteristics of the image reconstruction process. Moderate to low energy tomographic techniques are important for the future of medical imaging, especially for safe in vivo data acquisition and for prolonged continuous monitoring. Standard computer tomography X-ray scan (CT), positron emission tomography (PET) or magnetic resonance imaging (MRI) are not applicable for prolonged studies given their dimensions, the patient confined conditions and the negative effects of continuous exposure of their excitation methods. Currently, two low energy methods are the most studied: echotomography and electrical impedance tomography. These techniques are non-invasive in *sensu stricto*, given that emitters and sensors need to be only located on the surface (skin) to get the internal distribution of acoustic or electrical impedance that after is translated to the medical image. The echo/electrical tomography techniques have been proven to be successfully applied in many cases reported in the literature. From the medical perspective, these techniques are pivotal to providing functional information to the patient and are especially useful in cardio-pulmonary monitoring [14,15,16,17].

The problem of the low-energy tomographic techniques is their inferior image resolution with respect to standard CT. Life presents tissue and organs with complex structures and includes electrochemical and mechanical activity, increasing the challenge of obtaining images with higher quality. One aspect to consider is the sensor interface that is limited to a very small set in current low-energy tomography (rarely over a hundred sensors) but can reach over 50 thousand sensors, for example in the case of PET. Therefore, the number of measurement points variate in several orders of magnitude between standard tomography scanning techniques and echo/electrical tomography. Another aspect to consider is that in traditional tomography via scanning, the sensor position is controlled and surrounds the subject by circular sections (slices) or continuously in spiral CT data acquisition. For echo or electrical tomography, it is common to have a robust sensor-skin interface, implying that the computational model used for image reconstruction needs to be tuned for each individual and needs to account for the volume variations that change sensor spatial positioning. It is possible to obtain a stable sensor position in echo/electrical tomography, immersing the patient in a tank system for measurement, but it will reduce the applicability of these methods for long term monitoring. The skin tissue interface is a natural barrier of the body, given its layered cell composition. From the electrical point of view, live tissue responds to excitation frequency. Variations in the signal frequency are correlated to signal penetration in the body and to the tissue structure. International standards designate limits for the energy of the excitation signal with respect to the frequency, for safety reasons. These elements, complex tissues and safety energy limits, motivate the design of a device to explore waveforms with respect to their propagation characteristics in different body tissues: skin, bones, lungs and particular muscles.

With the generation of digital re-configurable devices for mixed signals using FPGA, fast, parallel and coordinated data acquisition is the basic advantage [18]. For real-time monitoring in tomography, FPGA is an essential element, given their prototyping capabilities, and as intermediary design for application-specific integrated circuits (ASICs). For example, in the ASIC based active electrode system of [19], the central hub is controlled by an FPGA. Another application of FPGA is as a hardware accelerator of computation routines. A semi-parallel potential and current excitation emitter were developed using FPGA as a hardware accelerator [20], for FFT calculations and signal filtering. Further applications can include on-line non-linear analysis like mutual information [17], acceleration of reconstruction using iterative algorithms and also artificial intelligence algorithms, for example, neural networks.

One of the specific objectives of this work is to incorporate waveform reconfiguration capabilities to the electrical tomography device, continuing the preliminary work of [21,22]. That will allow us to experiment with different modulations of the emitter waveforms, and it incorporates specialized architectures in its design, considering FPGA reconfigurable hardware and a microcontroller-based network gateway for *internet of things* (IoT) interoperability. The technical improvements achieved in this work include:

- Precise time synchronization of the parallel execution of excitation and sensing processes.
- Real-time reconfiguration of excitation signals.

In this work, we analyze the EIT input/output data, incorporating the ideas of impedance spectroscopy [23] and bioimpedance [24]. This topic is broadly studied in electrical impedance tomography spectroscopy [25].

For the EIT image, we test a standard reconstruction method under a tomographic configuration with dedicated electrodes for excitation and sensing. The advantage of these design characteristics is that it allows for the future implementation of online system identification and optimization, for example, to obtain a tissue impedance model and to calculate the optimal waveform for the measurement target. In the literature, there are many examples of EIT devices using FPGA technology [19,20,26]. Their use represents a great advantage in mixed-signals EIT and their real potential is still underutilized. That is the case with composite devices with many heterogeneous subsystems, where our current design is a representative example: a mobile solution for multi-hour measurement in the field of telemedicine, it comprises an EIT measurement system, a combination of sensors and a wearable vest. This variety of elements are considered in the design of the project where this work form part is the most distinguishing feature and novelty in relation to existing EIT+FPGA solutions.

In this work, we study an electrical tomography (ET) image device with the reconfiguration of the emitter for on-line customization of excitation signals. The device is conceived for medical monitoring and in vivo data acquisition and is implemented in FPGA hardware for real-time signal acquisition and a microcontroller SoC to provide escalation of functionality in IoT environments.

## 2. Materials and Methods

### 2.1. Impedance Modeled Tomography

Impedance based modeling methods are broadly used in different fields, from circuits, acoustic and biosignals to civil engineering structural analysis and robotic control. The general idea of impedance is to compare an input flow and an output pressure [27]. For ET measurements, the flow is electrical current I(t) and the pressure is electrical potential V(t). Impedance can be modeled using the Laplace transform of the ratio of measured potential over the excitation current, that is the transfer function of the system. Impedance can be measured on-line using for example power spectral density [28] and fast Fourier transform estimation techniques in modern data acquisition equipment [29,30].

**Figure 1:**
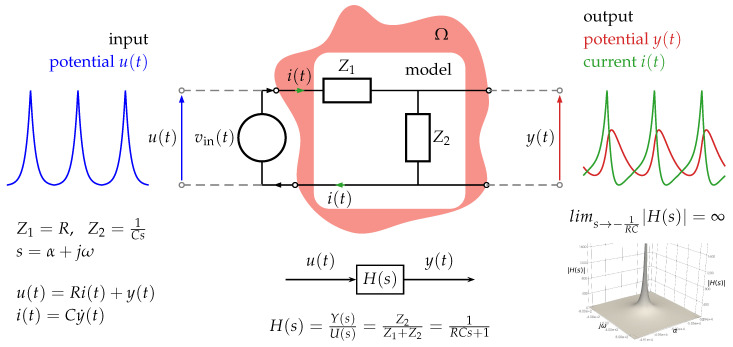
System Response and Impedance in a SISO system.

Figure 1 presents the general concept of impedance modeling in a single-input single-output (SISO) system. Input-output signals u(t) and y(t) are measured to estimate the system response with respect to a selected linear model in *s*-domain $H\left(s\right)=Y\left(s\right)U{\left(s\right)}^{-1}$, where U(s) and Y(s) are the corresponding Laplace transformed counterparts of u(t) and y(t). The variable s∈C is usually described as s=α+jω where α is the attenuation coefficient and jω the frequency coefficient.

As an example, if we consider that the system consists of two impedance elements connected in series, then for any selection of impedance elements $Z_{1}$ and $Z_{2}$, the model will be described by the ratio of the impedance in the output with respect to the total impedance in the input $H\left(s\right)=Z_{2}{\left(Z_{1}+Z_{2}\right)}^{-1}$. If $\left(Z_{1},Z_{2}\right)$ consists of a pure resistor *R* and a pure capacitor *C*, then $H\left(s\right)={\left(RCs+1\right)}^{-1}$. This transfer function has a single pole located at $s=-{\left(RC\right)}^{-1}$, representing an exponential decay response. As the single pole is real and negative, the system response is asymptotically stable. The corresponding time domain equation is:

(1) $$\dot{y}\left(t\right)={\left(RC\right)}^{-1}\left(-y\left(t\right)+u\left(t\right)\right).$$

For a linear system, we only need to know the transfer function of the impulse response $h\left(t\right)=L^{-}1\left\{H\left(t\right)\right\}$ (with L the Laplace transform operator), to obtain the response of a particular input signal u(t) by convolution:

(2) $$y\left(t\right)=\int_{-\infty }^{+\infty }u\left(t\right)h\left(t-\tau \right)d\tau .$$

For system (1), we can obtain a general analytic solution for an input u(t), using (2), given that the impulse response is $h\left(t\right)={\left(RC\right)}^{-1}e^{-\frac{t}{RC}}$:

(3) $$y\left(t\right)=y\left(0\right)e^{-\frac{t}{RC}}+{\left(RC\right)}^{-1}\int_{0}^{t}u\left(\tau \right)e^{-\frac{\left(t-\tau \right)}{RC}}d\tau$$

In Figure 1, the input wave pattern used is $u_{\exp}\left(t\right)$, defined as:

(4) $$u_{\exp}\left(t\right)=Ae^{-B\left|2ft-1\right|},\;0<t<\frac{1}{f}.$$

The parameters (A,B,f) in (4) represent the excitation amplitude A>0 in [V], the exponential factor B>0 and f>0 the desired fundamental frequency of the input wave in [Hz].

**Figure 2:**
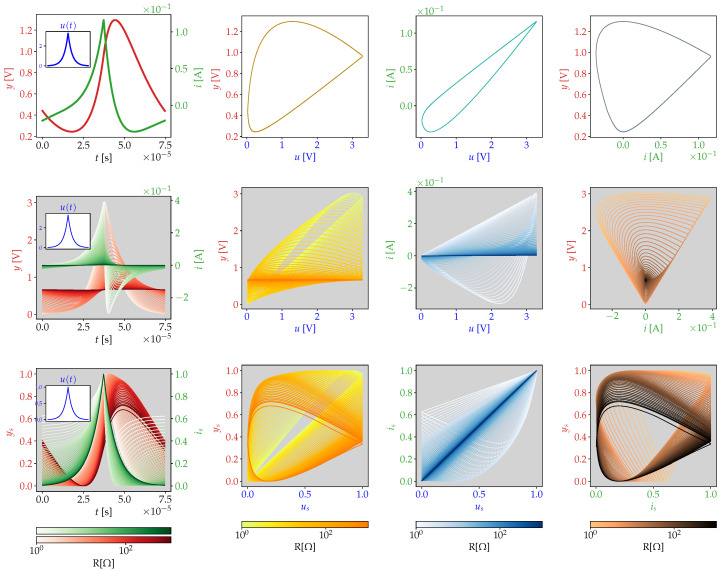
System response phase portraits for an input generated by (5) using $C=10^{-6}$ [F] and different values of *R* [Ω]. First column: current *i* and potential *y* for the excitation signal *u*. Second column: (u,y) plane. Third column: (u,i) plane. Fourth column: (i,y) plane. First row: R=20 [Ω]. Second row: R∈[1,1024] [Ω]. Third row: R∈[1,1024] [Ω] and variables scaled to [0,1].phase portrait

An even more general view is to consider the phase-portrait of potential and current (V,I). In ET, the phase portrait captures the oscillatory behavior of the measurement process. The analysis of the plane (V,I) has the advantage of accounting also for nonlinear dynamics. In the standard case of sine wave excitation signals, the phase portrait is ellipsoidal, where the shape of the ellipsoid represents the phase shift between *I* and *V*, this method has been used for example in impedance spectroscopy [23]. The plane (V,I) is used to identify defaults in circuits, comparing a reference signature $\left(V_{c}\left(t\right),I_{c}\left(t\right)\right)$ with the response of the circuit under test $\left(\hat{V}\left(t\right),\hat{I}\left(t\right)\right)$ in the analog signature analysis [31,32]. A phase-portrait example for the circuit model of Figure 1 is presented in Figure 2, we account for a general method to numerically estimate the response of the system (1) without confine the underlying model to be linear and therefore without using (2). To simulate the circuit, we use a Fourier series approximation of the input ${\hat{u}}_{\exp}\left(t,k\right)$, where the number of cosine terms considered is given by k∈N:

(5) $${\hat{u}}_{\exp}\left(t,k\right)=\frac{A}{B}\left(1-e^{-1}\right)+2AB\sum_{n=1}^{k}\frac{{\left(-1\right)}^{n}-e^{-B}}{B^{2}+{\left(\pi n\right)}^{2}}cos\left(2\pi ftn\right).$$

In Figure 2, numerical integration of the system (1) was performed. This system becomes stiff under the input generated by (4) therefore the Radau method was used. The input u(t) was approximated with ${\hat{u}}_{\exp}\left(t,k\right)$ using (k=200) cosine terms. Giving (1), we can appreciate that the phase portrait can be represented by $\left(y,\dot{y}\right)$. The three rows of Figure 2 displays the behavior of the system under variable resistor values, maintaining constant the capacitor in $10^{-6}$ [F]. The first row presents the system response for a single value R=20 [Ω]. The second row presents the phase portraits for different pairs of the variables (u,i,y), to express the magnitude change under variable resistance. In the third row, the variables are scaled to the interval [0,1] to display the shape change. We can appreciate clearly two different behaviors at lower and higher values of *R*.

ET data acquisition can be considered similar to the impedance response concept of Figure 1, but in a multi-input multi-output (MIMO) system. The measurement process in ET sample a distribution model σ:Ω→R under study. Continuously, excitation signals $u_{1},u_{2},\ldots ,u_{M}$ are emitted on the surface ∂Ω of the unknown system. At the same time the electrical potentials $y_{1},y_{2},\ldots ,y_{N}$ are sensed in different locations of ∂Ω to get a representative sample of the surface system response to excitation. This MIMO system has a transfer function matrix H(s), that in general connects input with output following $Y_{N\times 1}\left(s\right)=H_{N\times M}\left(s\right)\;\cdot \;U_{M\times 1}\left(s\right)$. The ET measurement process is designed to acquire sufficient surface potential data to infer the internal conductivity distribution Ω of the unknown system, the subsurface “image”. A concise overview of electrical impedance tomography imaging for organic tissue is presented in [33].

### 2.2. ET Platform Design for IoT in Healthcare

This work belongs to a large and complex project that aimed to create a prototype for an innovative mobile cardio-respiratory monitoring device, described in the Introduction. These types of mobile solutions, which are not currently available, require solving a number of research problems involving design, testing and performance with miniaturization and mobility.

**Figure 3:**
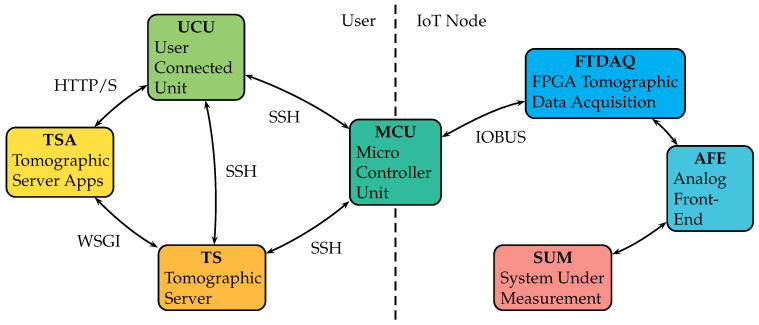
ET/IoT Healthcare platform, building blocks.

We have specific requirements for the design, two of them are mobility and motoring capabilities and therefore IoT is a solution to the architecture design. This inclusion has deep implications, for example, the data need to be stored and sent to intermediary servers given the limited memory resources of IoT devices. The use of wireless/IoT/server technology is presented to describe the design requirements and constraints. To list the general aspects of the ET platform, an overview of the system is displayed in Figure 3. The main goal of this ET/IoT platform is to provide monitoring and support for networked healthcare.

We consider a design separated into fundamental elements for IoT-devices interoperability. It is a heterogeneous computing platform considering an XC3S1400AN FPGA device with 1.4M logic gates from Xilinx, a quad-core ARM Cortex based SoC microcontroller unit (MCU) from Broadcom that enables IoT wireless connectivity, a standard computing server (TS) and user connected units (UCU). The digital design for the FPGA device was developed in VHDL and in the MCU, the tomographic application programming interface (TAPI) was programmed in Python language.

In the top-left of Figure 3, the user interacts with the platform using a UCU for example a Laptop, Phone, Tablet with wide area network (WAN) access. The platform will provide services for two types of users, with the roles of client and expert. A client can have access to services exposed in the TSA block, using a web interface for server-side applications. An expert can connect directly to the tomographic server (TS) where raw data is available using secure shell communication (SSH).

In the TS block, the web applications are executed, in this case using the web server gateway interface (WSGI). Expert users can also have direct access to the MCU block, the microcontroller unit. MCU is an interface to the data acquisition system and a gateway for the IoT node. In the MCU block, the TAPI provides user-friendly, high-level programming functions to interact with the data acquisition block FTDAQ. The data acquisition is triggered by the MCU using default configuration or a user customized configuration via TS–MCU or UCU–MCU. Using the TAPI, the configuration is loaded in FTDAQ and the data of the experiment can be read back from the FTDAQ memory to the MCU and directly stored into the database of the TS.

A bidirectional (input-output) parallel bus (IOBUS) is conceived for simplicity between MCU and FTDAQ. The analog front end block, AFE, contains elements for the digital control, the mixed signals from the analog to digital and digital to analog conversion and the signal conditioning for the emitter and for the sensor part. Finally, the SUM block stands for the system under measurement and it is composed of the electrode interface and the measurement system that can be a test circuit, a phantom material tester, organic tissue, or a subject under study.

### 2.3. FTDAQ System Design

**Figure 4:**
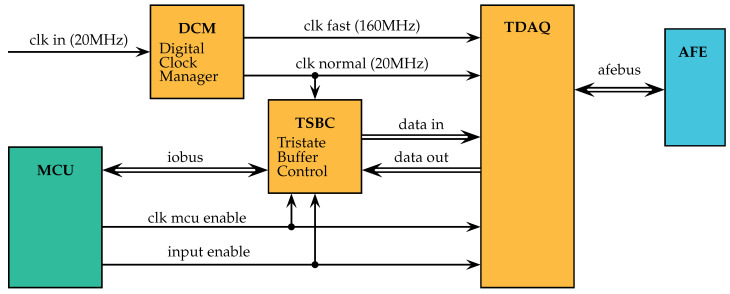
FTDAQ system design, top view. Double lines represent parallel connections.

The top view of the FTDAQ device is shown in Figure 4. Single lines are one-dimensional signals and double lines represent vector (parallel) signals. FTDAQ considers a specific but parameterized design. It uses two clocks, one fast for data acquisition at 160 MHz and one slow at 20 MHz for communication with the MCU. It has the following functionalities:

- Store a periodic excitation waveform in a RAM device synchronized to a foreign, irregular clock enable signal from a MCU.
- To use as default excitation a set of waveforms are stored in a ROM.
- Configure the emitter parameters (output frequency).
- Trigger and drive the excitation signal using a precise and fast clock.
- Synchronize sensors to acquire the response signal at the same clock that the excitation signal.
- Store the acquired signal in RAM at high speed using the fast clock.
- Write back to MCU device from RAM using a slow clock to synchronize with the MCU.

In the top view of Figure 4, the interface of the main elements is presented. The base clock of the FPGA is not used directly but routed to a digital clock manager (DCM) block where a controlled and simultaneous generation process of the two output clock signals is made. The maximum practical clock speed of the FPGA device used (a Xilinx XC3S1400AN) is around 300 MHz but modern FPGAs can arrive up to 1 GHz. The high speed clock is required for data acquisition, as one of the bottlenecks of the process is the speed of the digital to analog and analog to digital converters. The slow speed clock is used to communicate with the MCU. The MCU used is a Broadcom SoC, running Linux. The advantages of programming an MCU with OS are their simplicity and generality: standard Linux programming, software and networking functionality are available out of the box, providing an ideal WAN gateway for the IoT node. The disadvantage is that the signals generated can have very high latency from time to time, the expected behavior given that the kernel orchestrate the process priorities in the whole system. The latency problem for communication with the FPGA is solved in the traditional way in the TAPI: Two control signals are generated in the MCU, a clock enables and a input enables, to allow the FPGA to use their internal clock and not the irregular clock signal generated in the MCU. The objective of these two control signals is to control an input output parallel communication bus, the iobus. The iobus is the most important bottleneck of the system and is a limitation of the MCU used. The tristate buffer control block (TSBC) is in charge of controlling the direction of the bus, driving to high impedance to block the bus in one direction at a time. Thanks to TSBC, the tomographic data acquisition block (TDAQ) is isolated from the details of the communication interface of the MCU. The TDAQ uses the slow clock to communicate with the TSBC-MCU and the fast clock to control the data acquisition process and the communication with the Analog Front End peripherals (AFE).

### 2.4. Emitter Design

**Figure 5:**
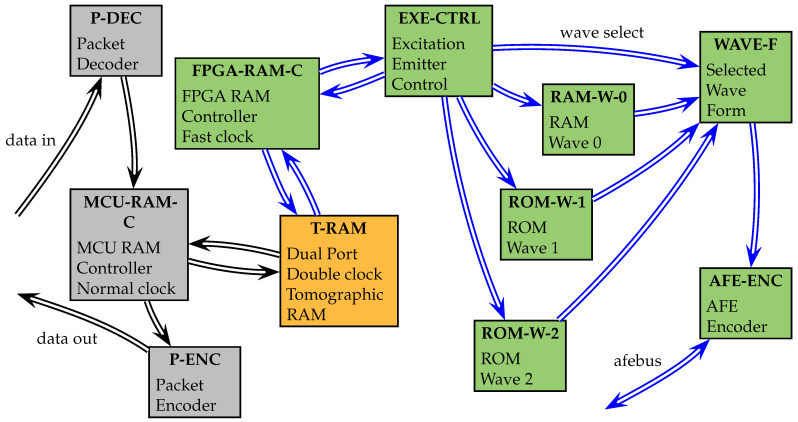
FTDAQ Emitter design part, TDAQ device. Blue arrows represent high speed driven connections, black arrows are normal speed driven connections.

In Figure 5, we present a simplified diagram of the emitter design. It presents the implementation of the block TDAQ of Figure 4. The color of the double-lined arrows represents the speed of the clock that drives the process. Black arrows are signals controlled with the 20 MHz clock and correspondingly blue arrows are signals controlled with the high speed clock of 160 MHz. The color of the blocks represents the same fact, gray blocks for standard speed and green blocks for high speed. The gray blocks are related to communication operations with the MCU, and the green blocks are related to the emitter data flow and processing.

The exception is the yellow block T-RAM, which is a crucial device in the design, because operates in parallel in two clock domains. Vector data input (“data in” from Figure 5) represents a packet of data, that enters into the packet decoder block (P-DEC). Each incoming packet is composed of an operation code and a payload. The main operations are:

1. Set read address for T-RAM.
2. Set write address for T-RAM.
3. Set data to write for T-RAM.

In the MCU RAM controller block (MCU-RAM-C), the read address, write address and the data to write are stored in registers. Depending on the code and on the current state, the MCU-RAM-C configure read and write addresses in the T-RAM and provide the enable signals for reading the T-RAM and writing the result back to data registers. Depending on the case, the registered are loaded into the Packet encoder block (P-ENC) where payload output codes are merged into an output packet (data out). The dual-port double-clock tomographic RAM (T-RAM) is mapped as:

- The first 100 addresses (0–99) points to the re-configurable excitation waveform data.
- The next 10 addresses (100–109) points to the tomographic data acquisition parameters.
- The rest 65,425 addresses (110–65,535) points to the sensed tomographic data.

Helper high level functions for reading and writing to T-RAM are available in the TAPI of the MCU. The FPGA RAM controller block (FPGA-RAM-C) manages the reads and writes from the T-RAM. In this work we are focused on the emitter, therefore the sensing procedure will be not analyzed. For the emitter, the FPGA-RAM-C block reads the T-RAM every time that the tomographic data acquisition parameters are updated in addresses 100–109. The emitter parameters are loaded for FPGA-RAM-C into the excitation emitter control block (EXE-CTRL). The base parameters are:

- The wave output sample clock, that controls the speed of the output wave.
- The wave selected code, that selects which waveform to load: RAM-W-0, ROM-W-0, or ROM-W-1.

The read-only memory waveforms of blocks ROM-W-0 and ROM-W-1 store default waveform patterns. RAM-W-0 load the data directly from T-RAM, via the EXE-CTRL block. The selected waveform block (WAVE-F) is directed to the AFE encoder block (AFE) to write the excitation sample to the DAC register and complete the excitation process for this particular sample.

### 2.5. Mobile Tomographic System

The aim of this research project is to develop a solution concerning a mobile tomographic system for 3D imaging and surface monitoring using body surface potential mapping. From the digital design, presented in the previous subsections, we considered an external microcontroller that communicates with the base FPGA. Those communications were made with a specific bus where simple communication capabilities were provided. The development and testing of the interface were time-consuming and the priority schedules of the microcontroller OS do not contribute to fast data transmission from the FPGA memory to the microcontroller memory. Given the experience acquired, we have updated the digital design in the following manner:

- In our current prototype, we use a new technology that allows for software-hardware codesign. A system-on-chip Zynq 7020 from Xilinx, with FPGA+ and ARM processor, provides direct communication between the processor communication bus and the FPGA fabric.
- For the new development, we have explored standard communication buses (Wishbone and AXI). We have selected the AXI bus, reducing the complexity between programming user interfaces and the FPGA logic, and allowing for interoperability with respect to the available computing cores.

The operative design considers the following parameters in terms of power supply, battery, power consumption, operating frequency range, voltage/current amplitude, signal-to-noise ratio and architecture, such as:

- Excitation signal generation block—based on two high-speed DAC converters with a measuring shunt system and a 16-bit 25MSPS ADC converter, which together with the FPGA system creates digital feedback loops.
- Measurement block—a set of amplifiers and pre-filters multiplexed into 32 channels with a PGA gain control system and a 16-bit 25MSPS differential analog-to-digital converter.
- Body surface potential mapping block—A set of 102 active addressable measurement electrodes based on a signal matching and amplification unit.
- Power supply 5 V DC/Battery powered LI-Ion 3000 mAh, Energy consumption during measurement 2 W, sleep mode 0.1 W, frequency 50 kHz, Amplitude 500 mV p-p, current 100 μA. Signal noise ratio 90 dB.

### 2.6. Tomographer Design with Dedicated Electrodes

The implementation of the MIMO system for tomography described previously in Section 2.1 involves the use of excitation and sensing hardware that need to be mapped to the electrode interface located on the surface of the measuring element. A SISO measurement of excitation/sensing considers a quadruple of electrodes $q=\left(a,b,m,n\right)\in N^{4}$ where the first pair (a,b) denotes the excitation signal electrodes, and the second pair (m,n) references the sensing electrodes. For an ET system with *L* electrodes, the set of valid configurations is given by:

(6) Q(L)={(a,b,m,n)|a≠b≠m≠n,∀a,b,m,n∈[1,L]}.

The number of valid configurations is:

(7) $$\left|Q\left(L\right)\right|=\frac{L!}{(L-4)!}=\prod_{i=1}^{4}\left(L+i-4\right).$$

For every configuration quadruple in Q(L), the excitation and sensing signals need to be routed to their corresponding electrode pairs. That can be done using a bidirectional switch from 4 to *L* channels. In order to reduce the inclusion of extra hardware connections, we use the 4-channel multiplexed DAC/ADC available on the FPGA device board to develop a tomographer with (L=16) dedicated electrodes ($e_{1},\ldots e_{16})$, 4 of each type (a,b,m,n). That restricts the design to an even number of electrodes, then L=2M,M≥2,M∈N.

For a circle-shaped tank system, the electrodes were positioned in ring form, counter clockwise every 2π/L rad following the pattern a,b,m,n. The map between electrode type enumeration and global electrode enumeration is:

(8) $$q_{k}=\left(a_{k},b_{k},m_{k},n_{k}\right)=\sum_{j=1}^{4}c_{j}\;e_{4k+j-4},\;\forall k=1\ldots L/4,$$

where $c_{j},\;j=1\ldots 4$ are the elements of the standard basis for $Z^{4}$, $c_{1}=\left(1,0,0,0\right),\ldots ,$ $c_{4}=\left(0,0,0,1\right)$. Similarly, the dedicated electrode configuration set is defined by:

(9) $$Q_{d}\left(L\right)={\left\{\sum_{j=1}^{4}c_{j}\;e_{4k+j-4}\right\}}_{k=1}^{L/4}.$$

The configuration set $Q_{d}$ of (8) allows for $|Q_{d}{\left(L\right)|=\left(L/4\right)}^{4}$ measurements, therefore the ratio with respect to the original space following (7) will reach the minimum value of $4^{-4}$ for larger *L* (≈0.39% of the original space):

(10) $${lim}_{L\to \infty }\frac{|Q_{d}\left(L\right)|}{\left|Q(L)\right|}=\frac{1}{4^{4}}.$$

In the case of 16 electrodes, $|Q_{d}\left(16\right)|=256$, less than 0.6% of the original space of |Q(16)|=43,680 configurations. Is interesting to compare these values with the EIT adjacent and opposite measurement strategies, as described for example in [34] accounting for L(L−3) electrodes with a ratio of ${\left(L^{2}-3L+3\right)}^{-1}$ with respect to |Q(L)|. The ratio, in this case, approaches zero as L→∞.

For the number of measurements in adjacent and opposite strategies, we consider the complete circle scan (2π rad) for the L(L−3) configurations and not only the independent measurement configurations (that will account L(L−3)/2 in the case of adjacent) because we want to take into account:

- Heterogeneous, non equidistant electrode positioning on the surface ∂Ω.
- Temporal variations on the shape of the object under study Ω(t) e.g., tidal volume variations.
- The acquisition, collection and processing of redundant data for research on signal and image processing.

When there is a need to sense a large 3D area, more efficient measurement schemes can be selected to scan the available 3D directions for excitation and sensing. A well-designed data acquisition strategy can be deployed to avoid the measurements being confined to the electrode plane directions in the case of electrode ring configuration.

## 3. Results

### 3.1. SISO Data Analysis

The goal of the SISO data analysis is to test the waveform-reconfigurable emitter with a minimal quantity of elements. To achieve this goal, the SISO model presented in Section 2.1 was used to acquire data from the tomographer with excitation frequency f=11.43 [kHz]. The configuration consists on a pure resistor of R=218.6[Ω] for $Z_{1}$ and for $Z_{2}$ a capacitor of 1[μF]. We explore the effects of selecting different waveforms for the system in Figure 1. For the SISO data acquisition, The waveforms are designed to share the same amplitude and frequency but carry different energy. The energy *E* of the signal x(t) is defined by $E=\int_{-\infty }^{\infty }{\left|x\left(t\right)\right|}^{2}dt$.

A compilation of the designed waveforms is presented in Table 1, with the fundamental frequency *f* as a parameter and with a waveform range between 0 and 1. The first column of Table 1 displays the quantized version of the waveform as is loaded in the T-RAM. The second column is the name index k∈{A,B,C,D,E,F} that identifies every waveform. The third column of the table shows the waveform definition $u_{k}\left(t\right)$ with frequency as a parameter. In the fourth column, the energy $E_{k}\left(f\right)$ is displayed for every case except B, which is a function composed of adding two waveforms and scaling them to [0,1]. The fifth column presents the numerical computation of the energy ${\tilde{E}}_{k}\left(f\right)$ when f=1.0, calculated with five significant digits. The signals are ordered by increasing the energy level. The energy definition for the special case B is given by:

(11) $$E_{B}\left(f\right)=\frac{1}{b^{2}}\left(E_{F}\left(f\right)+E_{A}\left(f\right)+\frac{a^{2}}{f}+2\int_{0}^{1/f}\left(u_{F}\left(t\right)u_{A}\left(t\right)-au_{F}\left(t\right)-au_{A}\left(t\right)\right)dt\right),$$

where $a=\min\{u_{F}\left(t\right)+u_{A}\left(t\right)\}$ and $b=\max\{u_{F}\left(t\right)+u_{A}\left(t\right)\}-a$ are used to scale the waveform to [0,1].

From Table 1 we can observe that for constant frequency and with the definition of $u_{k}\left(t\right)\in \left[0,1\right]$ is possible to control the signal energy varying the amplitude A>0 as the energy of $Au_{k}\left(t\right)$ is $A^{2}E_{k}\left(f\right)$. Similarly, if the amplitude is constant we can control the energy varying only the frequency.

**Figure 6:**
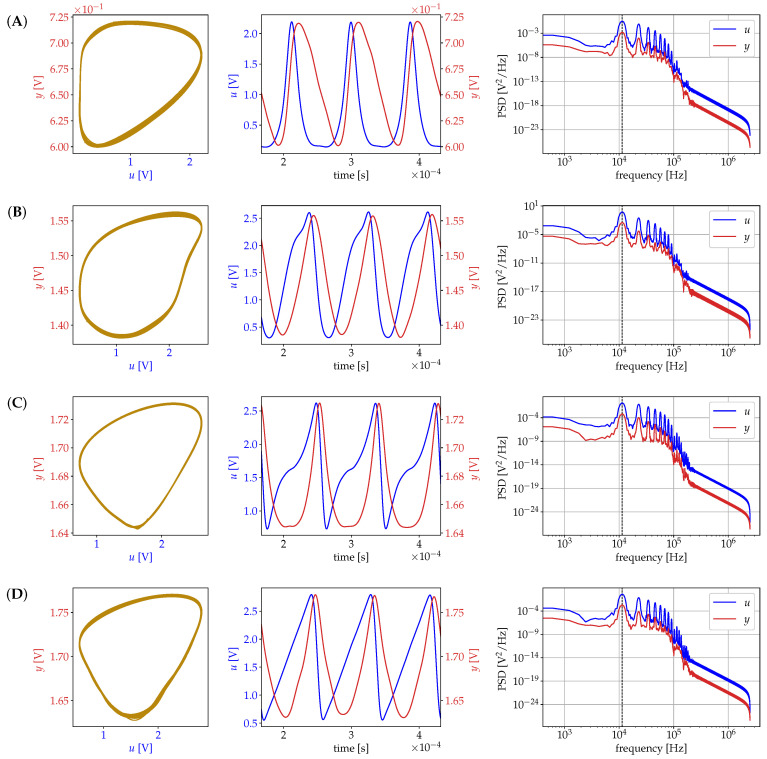

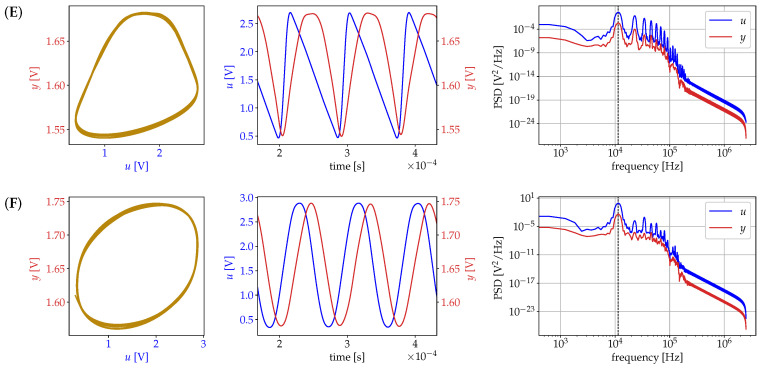
Measured excitation response for six different waveforms. Rows from (**A**) to (**F**) correspond to the waveforms described in Table 1. First column: phase portrait (u,y). Second column: time series of excitation (blue) and response (red). Third column: log-log plot of power spectral density of excitation (blue) and response (red).

In Figure 6, we measure the response of the SISO model of Section 2.1 for the six waveforms of Table 1. The acquired signals were processed using a digital low-pass filter. The first column of Figure 6 presents the phase portrait (u,y). It displays the oscillatory behavior of the acquired data output with respect to the input, similar to the in silico results of Figure 2. For the corresponding timeseries, a snapshot of duration 3/f is presented in the second column. Finally, the power spectral density (PSD) of the signals is presented in the third column. The effect of the low-pass filter is appreciated from around $10^{5}$ [Hz].

If we compare waveforms A–E with the “sine” wave F we can appreciate that “sine” has a constant lag between *u* and *y*, but the rest of the waveform has variable lag. Consequently, the PSD of “sine” present their first peak in the fundamental frequency, but the following peaks are weak. The rest of the signals has well marked high frequency harmonics. This is of particular interest in the case of organic tissue given its frequency-dependent response to electrical excitation.

### 3.2. MIMO Data Analysis for Electrolyte Tank Measurements

We use the configuration described in Section 2.6 with $Q_{d}\left(16\right)$ following (9). For the $|Q_{d}\left(16\right)|=256$ configurations, output potentials $Y_{N\times W}$, were measured with $N=|Q_{d}\left(16\right)|$. The number of consecutive samples on the time window was defined by W∈N.

In [35], the concept of potential profile for EIT was briefly described. To display the *N* configurations, we first sort the measured potential value in $Y_{N\times W}$ to set up a reference ordering: $y_{1,w}\leq y_{2,w}\leq \cdots \leq y_{N,w}$ for 1≤w≤W. This ordering is a better choice than the default arbitrary ordering of the output potentials used in many EIT systems. In this way, we can track the combinatorial changes in the ordered indexes of a reference measurement with respect to another. Generally, the reference is set for a homogeneous material configuration.

Also in [35], the analysis was extended to classify the potential response given the axial symmetry of the tank systems with equidistant electrodes. The metric used was the ring distance between the excitation electrode pair (i,j) for a tank of *L* electrodes:

(12) $$d\left(i,j\right)=\min\left(|i-j|,L-|i-j|\right)\;,\;\;\;i\in \left[1,L\right],\;j\in \left[1,L\right],\;i\neq j.$$

**Figure 7:**
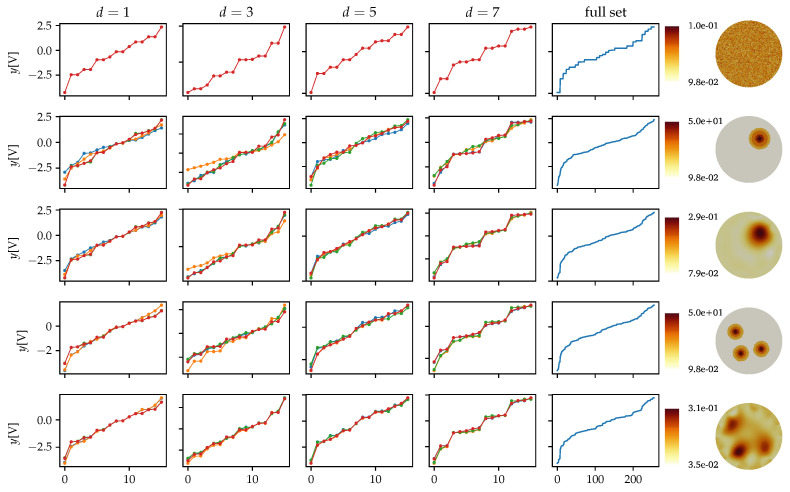
Potential profiles for different material distributions.

For $Q_{d}\left(16\right)$ configuration, there are only four possible potential profiles, classified by the ring distance of the excitation electrodes (d=1,3,5,7). Every profile consists of 16 configurations, that implies that there are only 64 unique responses for the 256 measurements in the homogeneous case. Modifying the material distribution with inhomogeneity will break the axial symmetry on the measurement and will produce permutations on a local scale, allowing us to observe the effect of the inclusion in the potential measurement in Figure 7.

**Figure 8:**
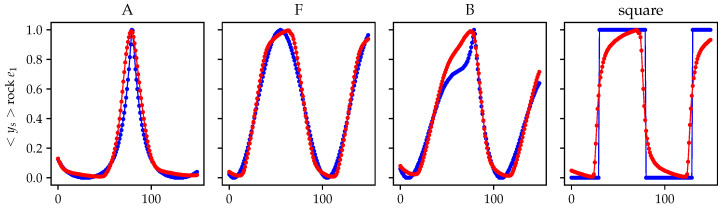
Scaled mean response measured ($<y_{s}>$, red) with respect to excitation waveforms (blue) for a rock inclusion near first electrode $e_{1}$.

**Figure 9:**
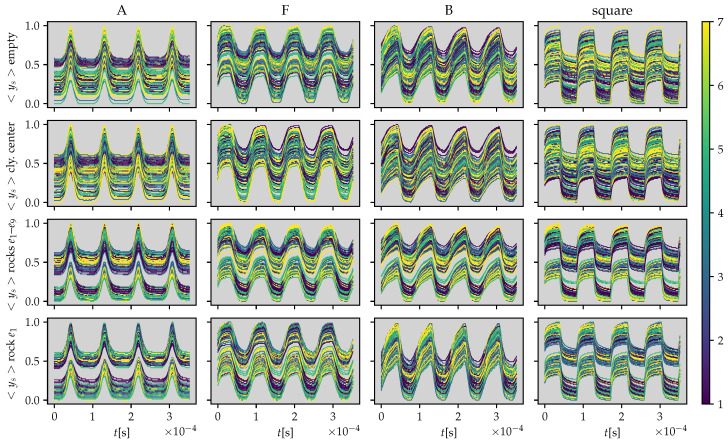
Measured responses with respect to excitation waveforms for four different material distributions. The colormap represents the ring distance between the excitation electrodes.

In Figure 8, a comparison of the excitation waveform and mean response $<y_{s}>$ for the 256 configurations are presented for a rock inclusion near the first electrode $e_{1}$. The full measurement set is presented in Figure 9 where each row represents four material distributions: (i) homogeneous (“empty”); (ii) a cylinder located in the center of the tank; (iii) two rocks located near the first and the ninth electrode; and (iv) a rock located near the first electrode. The timeseries are mapped to a color that corresponds to their ring distance (12) allowing to observe the potential variations with respect to the electrode distance and the potential variations with respect to the material distribution.

**Figure 10:**
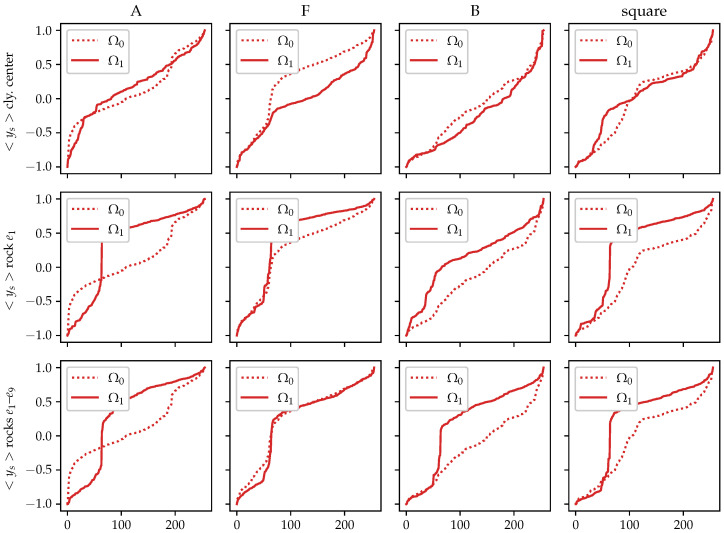
Potential profiles of measured responses for some material inclusions.

In Figure 10 the potential profiles of acquired data are presented, comparing the homogeneous (“empty”) measurement $\Omega_{0}$ with respect to the elaborated material distribution $\Omega_{1}$ for each case of Figure 9.

### 3.3. Tomographic Imaging

The purpose of presenting MIMO data for EIT tank measurements in the previous subsection was to underline the possibilities of modeling electrical tomography as a system identification problem. In this work, we focus on the design of a general method of response acquisition, but we maintain the tomographic imaging static. The EIT measurements in an electrolyte tank system is an important phase in the development of tomographic devices and methods and it is an element continuously present in the EIT literature, for example, in the recent works [36,37]. The use of tanks is fundamental for modeling and testing tomographic algorithms, given that the shape of the surface and the electrode locations are stable. This is crucial for measurement, and is an important step to continue further with more complex scenarios and dynamic shapes, as in the case of the human chest during respiration.

**Figure 11:**
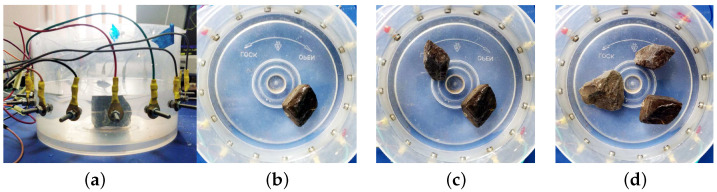
Tank setups for data acquisition. (**a**) Front view with electrode detail and rock in the center. (**b**) Rock in front of electrode $e_{9}$. (**c**) Rocks in front of electrodes $e_{1}$ and $e_{9}$. (**d**) Rocks in front of electrodes $e_{3}$, $e_{9}$ and $e_{13}$.

**Figure 12:**
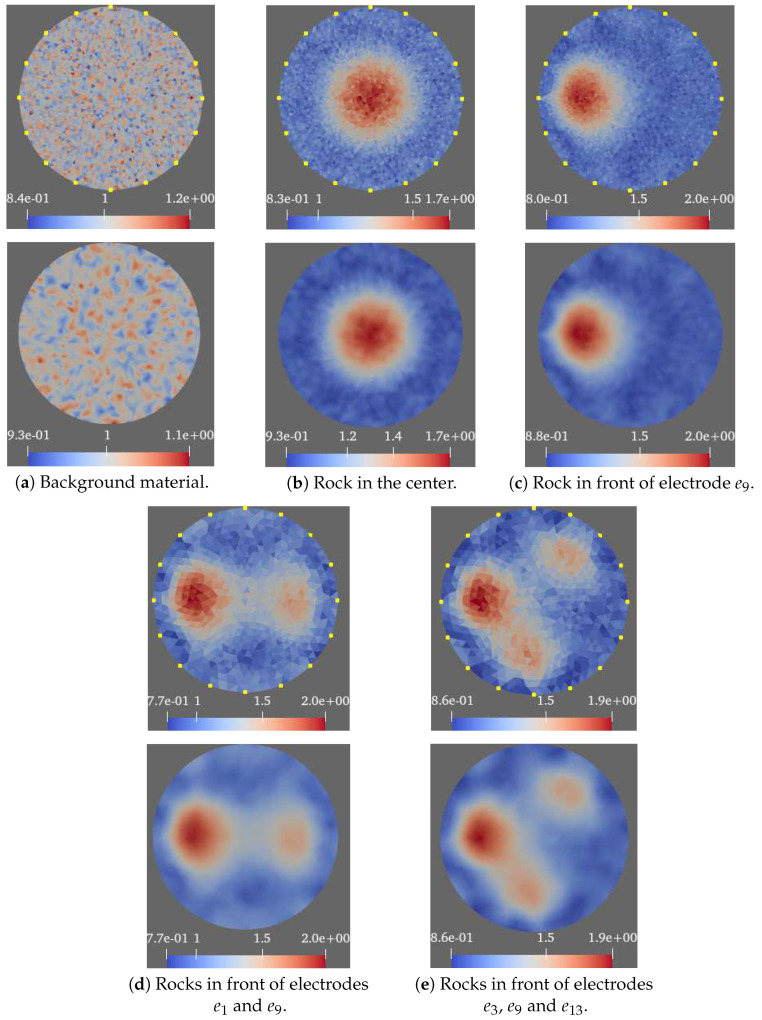
Tomography image for configuration $Q_{d}\left(16\right)$ for the selected material distributions.

In Figure 11, the tank setups are presented. They are composed of some small rocks presented in different configurations. In Figure 12 the tomographic imaging is presented for these setups. Note that the resulting images are rotated counter clock wise with respect to the original configurations (electrode $e_{1}$ is located in 0 degrees and the following electrodes are located at equidistant positions, in counter clock wise direction). For each set of material distributions, two images are presented one sharp and another fuzzy. The sharp image represents the delta conductivity image, where the resulting conductivity values are directly mapped to every triangular element in the mesh.

The yellow points in the boundary represent the electrode positions. The mesh used consist of over 12k elements, where the triangle area has a small standard deviation (triangles are very similar), therefore operations from triangle elements to points can be approximated only by connectivity (neighborhood) without the incorporation of weights based on triangle area. The fuzzy image is the result of the projection of the delta conductivity values from the individual value of each triangle to their vertices. In particular, the projection consists of the mean value of all the neighbor elements to a point. The result of this projection is a low pass filter.

The method used for calculating the difference conductivity image $\Delta \sigma =\sigma_{1}-\sigma_{0}$ is Gauss-Newton with regularization parameter λ=0.01. This is one of the many methods to estimate a solution of the inverse problem in electrical tomography and probably the best first option to use when data from a new system is provided. The conductivity image is calculated setting the linear approximation that maps material conductivity σ into surface potentials *v*:

(13) $$\left(\sigma_{1}-\sigma_{0}\right)=-J^{†}\left(v_{1}-v_{0}\right),$$

with $J^{†}$ the pseudo-inverse matrix for the Jacobian *J* and is calculated by:

(14) $$J^{†}={\left(J^{T}J+\lambda \sqrt{\mathrm{diag}(J^{T}J)}\right)}^{-1}J^{T}.$$

It is important to note that:

1. The Jacobian *J* is required as an input parameter for solving the inverse problem using a linear approximation as appear in (13) and (14).
2. The Jacobian *J* can be estimated, for example, solving the forward problem using the finite element method for the background material distribution.
3. A Jacobian element in a finite element method model represents the sensitivity of the electrical potential in the electrode nodes with respect to the variation of the conductivity in the mesh element.

Therefore, this methods requires the pre-computation of the forward EIT problem to estimate the value of the Jacobian, by the use of an arbitrary background conductivity $\sigma_{0}$. A detailed description of the estimation of the forward problem is available in the literature [38,39,40]. For a review of the assumptions of the EIT governing equation in live tissue (medium isotropy, internal current density neglected at high frequency excitation, magnetic effects negligible, capacitive effects negligible) we would like to refer to [41].

Calculation of figures of merit for evaluation of the reconstruction quality is not provided because the objective of the current imaging was to provide evidence that the excitation configuration used in this work, considering dedicated electrodes (disjoint sets of electrodes for excitation and sensing) can provide reconstruction in a similar manner to standard configurations. In the same way, the algorithm selected is classical and needs to be considered in any benchmark. Advanced modern algorithms using machine learning can be considered as a comparison, for example, Bayesian learning [42,43] or neural networks [8,44]. A comparative analysis of reconstruction strategy, excitation configuration and waveform modulation is part of further work in preparation.

## 4. Discussion and Conclusions

The project under which this research is structured consists of the construction of a mobile system with specialized hardware for measuring cardio-respiratory activity using electrical tomography and body surface potential mapping. Cardiorespiratory activity monitoring is the principal motivation for medical applications of EIT, from the pioneering work of Barber and Brown [45], combining ECG and EIT techniques [46]. Until now his work is relevant for EIT imaging, see, for example, [47]. EIT devices that measure biopotentials exist, as in the case of a wearable sensor for measuring EIT and ECG [48].

In this work, we present the elements of a tomographic design conceived for mobility and flexibility to experiment with the sensor process design. Electrical tomography is a moderate-to-low-energy tomographic method and it is well suitable for in vivo monitoring. The energy delivered by the tomographic system needs to be specified and controlled completely and that implies the analysis of the data acquisition system at the level of the waveforms, the focus of this work.

The concept of electrical tomography as an electrical perturbation (excitation) of the object under measurement is presented in Section 2.1. For a linear dynamical system, the object can be represented by the transfer function. This allows obtaining, for example, the response for a particular input signal using convolution. The response of the system can also be presented in a more general approach as a phase portrait, considering linear or non-linear dynamical systems.

The system architecture considers a microcontroller acting as an IoT gateway for online monitoring. A specialized bus is created to inter-operation between the microcontroller and an FPGA device, allowing the design of prototype measurement strategies with a high level of flexibility, especially in hardware and software codesign. The generation of a digital re-configurable device in the FPGA enables to experiment with different modulation of the emitter waveforms. An essential element for real-time monitoring in tomography, the FPGA can deliver a fast, parallel and coordinated data acquisition. Another advantage is the collection of excitation-sensing data in a precise manner, the FPGA device is designed to synchronize excitation and sensing processes.

Biopotentials, in particular action potentials, efficiently transmit information in the human body. These signals successfully traverse live tissue and the ability of propagation can be viewed as the product of evolutionary forces over the ages. Live tissue is complex media, and the ability to mimic some characteristics of action potentials can contribute to reducing the energy required to infer the media structure by an electrical perturbation on their surface. Given that this work is part of a project of EIT and body surface potential mapping for cardiorespiratory research in medicine, we are particularly interested in the nature of cardiac action potentials and their counterpart respiratory control of the muscles involved in respiration. International standards provide hard constraints for the current used to excite the human body, therefore is important to explore different modulations that can use lower energy levels to provide the same information that with usual sine-based waveforms.

The measured data are analyzed from different perspectives, presenting the time- domain timeseries of the responses and excitation, the frequency spectrum and their phase portraits for excitation and sensing data. The phase portrait is interesting because it shows the measurement process as an oscillator. From the phase space similar functionality can be gathered as in the case of I−V diagrams. The excitation and sensing signals correspond to input and output signals from a control theory perspective, and the dynamic estimation of the conductivity can be considered as a system identification problem. The waveform reconfiguration allows for fine-grained modeling signal energy and it can be further extended for online control, to fulfill the electrical current constraints for in vivo measurements.

In this work we analyze the incorporation of waveform reconfiguration capabilities to electrical tomography, following the classical approach used in impedance spectroscopy where not only sine waves, but square, random and chirp signals are used. Another interesting point is the design of a measurement strategy with dedicated electrodes, that can enable the study of specialized electrodes (different material and geometry) for sensing only or excitation only and it will be considered in further research. For analyzing the full set of measurement data, the concept of potential profiles was introduced. A sorted profile of electrical potential response to excitation is an interesting way of visualizing measurement variations given changes in the measurement process (different waveforms) or in the object under study (material distribution under measurement). The methods studied in this work can be easily adapted to the current EIT devices.

The tomographic image reconstructions are presented for the static case, using measurements in an electrolyte tank system. Ongoing work is taking into account a specialized soft-robotic phantom for dynamic image research [49] and lung biomechanics mimicry.

Further work considers the tomographic measurement of lung respiratory capacity and their correlations with the changes in lung impedance, in real time. Also, electrode configurations and excitation strategies for an extended quantity of electrodes in order to improve image resolution are envisaged as an extension of this work.

## Figures and Tables

**Table 1:** Waveform comparison with frequency as a parameter, sorted by energy level.

Wave	*k*	$u_{k}\left(t\right),\;u_{k}\in \left[0,1\right]$	$E_{k}\left(f\right)$	${\tilde{E}}_{k}\left(1\right)$
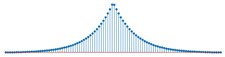	A	$\left\{\begin{array}{cc} \frac{e^{10ft}-1}{e^{5}-1}, & 0<t\leq \frac{1}{2f}\\ \frac{e^{10-10ft}-1}{e^{5}-1}, & \frac{1}{2f}<t\leq \frac{1}{f} \end{array}\right.$	$\frac{e^{10}-4e^{5}+13}{10f{\left(1-e^{5}\right)}^{2}}$	$9.8689\times 10^{-2}$
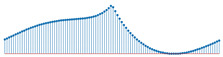	B	$\frac{u_{F}\left(t\right)+u_{A}\left(t\right)-a}{b}$, see (11) for (a,b)	see (11)	$2.8252\times 10^{-1}$
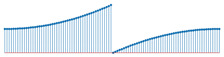	C	$\left\{\begin{array}{cc} 2{\left(ft\right)}^{2}+\frac{1}{2}, & 0<t\leq \frac{1}{2f}\\ -2{\left(ft\right)}^{2}+4ft-\frac{3}{2}, & \frac{1}{2f}<t\leq \frac{1}{f} \end{array}\right.$	$\frac{3}{10f}$	$3.0000\times 10^{-1}$
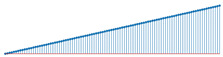	D	$ft,\;\;t\in \left(0,\frac{1}{f}\right]$	$\frac{1}{3f}$	$3.3333\times 10^{-1}$
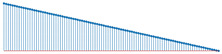	E	$1-ft,\;\;t\in \left(0,\frac{1}{f}\right]$	$\frac{1}{3f}$	$3.3333\times 10^{-1}$
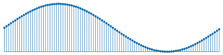	F	$\frac{1}{2}+\frac{1}{2}\sin\left(2\pi ft\right),\;\;t\in \left(0,\frac{1}{f}\right]$	$\frac{3}{8f}$	$3.7500\times 10^{-1}$
